# Differential Expression of Circulating miRNAs and Carfilzomib-Related Cardiovascular Adverse Events in Patients with Multiple Myeloma

**DOI:** 10.3390/ijms25147795

**Published:** 2024-07-16

**Authors:** Marwa Tantawy, Taimour Langaee, Danxin Wang, Samuel M. Rubinstein, Robert F. Cornell, Daniel Lenihan, Michael G. Fradley, Yan Gong

**Affiliations:** 1 Department of Pharmacotherapy and Translational Research, College of Pharmacy, University of Florida, Gainesville, FL 32610, USA; mtantawy@ufl.edu (M.T.);; 2 Center for Pharmacogenomics and Precision Medicine, College of Pharmacy, University of Florida, Gainesville, FL 32610, USA; 3 Division of Hematology, Department of Medicine, University of North Carolina, Chapel Hill, NC 27599, USA; 4 Division of Hematology and Oncology, Vanderbilt University Medical Center, Nashville, TN 37232, USA; frank.cornell@abbvie.com; 5 Cape Cardiology Group, Saint Francis Medical Center, Cape Girardeau, MO 63703, USA; 6 Thalheimer Center for Cardio-Oncology, Department of Medicine, Perelman School of Medicine, University of Pennsylvania, Philadelphia, PA 19104, USA; 7 Cardio-Oncology Working Group, UF Health Cancer Center, Gainesville, FL 32610, USA

**Keywords:** carfilzomib, cardiovascular adverse events, microRNA, cardio-oncology

## Abstract

This study investigates the association between circulating microRNA (miRNA) expression and cardiovascular adverse events (CVAE) in multiple myeloma (MM) patients treated with a carfilzomib (CFZ)-based regimen. A cohort of 60 MM patients from the Prospective Observation of Cardiac Safety with Proteasome Inhibitor (PROTECT) study was analyzed. Among these, 31 patients (51.6%) developed CVAE post-CFZ treatment. The Taqman OpenArray Human microRNA panels were used for miRNA profiling. We identified 13 differentially expressed miRNAs at baseline, with higher expressions of miR-125a-5p, miR-15a-5p, miR-18a-3p, and miR-152-3p and lower expression of miR-140-3p in patients who later developed CVAE compared to those free of CVAE, adjusting for age, gender, race, and higher B-type natriuretic peptide levels. We also identified three miRNAs, including miR-150-5p, that were differentially expressed in patients with and without CVAE post-treatment. Additionally, five miRNAs responded differently to CFZ treatment in CVAE vs. non-CVAE patients, including significantly elevated post-treatment expression of miR-140-3p and lower expressions of miR-598, miR-152, miR-21, and miR-323a in CVAE patients. Pathway enrichment analysis highlighted the involvement of these miRNAs in cardiovascular diseases and vascular processes. These findings suggest that specific miRNAs could serve as predictive biomarkers for CVAE and provide insights into the underlying mechanisms of CFZ-CVAE. Further investigation is warranted before these findings can be applied in clinical settings.

## 1. Introduction

Patients with multiple myeloma (MM) frequently have cardiovascular diseases, which can arise from various factors. These factors include MM-related issues like amyloidosis, non-MM-related conditions such as older age, diabetes, and obesity, and other aspects linked to MM treatment, such as anthracyclines, corticosteroids, alkylating agents, proteasome inhibitors, and B-cell maturation antigen (BCMA)-targeting chimeric antigen receptor T-cell (CAR-T) therapy [1,2,3,4]. Carfilzomib (CFZ), a proteasome inhibitor, is used to treat patients with relapsed and refractory MM. While effective, CFZ has been linked to significant cardiovascular adverse events (CVAE) [5,6,7]. Two meta-analyses showed that CFZ was linked to a higher incidence of CVAE, ranging from 8% to 18% [8,9], including heart failure (HF), hypertension, arrhythmia, and cardiomyopathy [3,10]. A recent study by Efentakis and colleagues [11] demonstrated that CFZ administration in male mice with cardiometabolic syndrome led to glucose and lipid dysregulation and cardiotoxicity, which can be mitigated by metformin and atorvastatin without exacerbating metabolic adverse effects. The study highlights the need for cautious use of CFZ in patients with diabetes, hyperlipidemia, and cardiovascular conditions, as well as close cardiac monitoring [11]. Identifying risk factors for CFZ-related CVAE (CFZ-CVAE) could enable preemptive ascertainment of at-risk patients who would benefit from prioritizing alternative agents or more aggressively managing cardiovascular risk factors [8,9].

MicroRNAs (miRNAs) are short, single-stranded non-coding RNA sequences consisting of 18-24 nucleotides that remain stable in peripheral blood. miRNAs play essential roles in gene regulation at the post-transcriptional level and are involved in different biological processes, including differentiation, development, proliferation, apoptosis, and necrosis [12,13]. miRNAs are also involved in the pathophysiology of cardiovascular diseases such as HF and myocardial infarction [14]. Different studies reported the association between chemotherapy-induced cardiotoxicity and the dysregulation of circulating miRNAs [15,16,17,18]. Research suggests that changes in miRNA expression profiles can serve as indicators of cardiac injury and dysfunction associated with cancer therapies [19,20,21,22]. miRNAs are detectable in various biological fluids, including blood and serum, making them accessible and minimally invasive biomarkers [23,24,25]. Monitoring specific miRNAs associated with cardiotoxicity can provide valuable insights into the early detection, prediction, and management of cardiac complications in cancer patients undergoing treatment. A recent study demonstrated that miR-34a expression showed a dose-dependent increase correlating with myocardial injury in mice treated with doxorubicin, highlighting its potential as an early marker for both immediate and delayed-onset doxorubicin-induced cardiotoxicity [26,27]. In this study, we aimed to identify differentially expressed circulating miRNA in the plasma of patients treated with CFZ at baseline and post-treatment that can be used to stratify patients for the risk of CFZ-CVAE and provide insights into the underlying mechanisms of CFZ-CVAE.

## 2. Results

### 2.1. Study Population

This study included a cohort of 60 patients with MM who were treated with a CFZ-based regimen from the Prospective Observation of Cardiac Safety with Proteasome Inhibitor (PROTECT) study [28]. The participants had an average age of approximately 66 years, with the majority being male (75%) and Caucasians (87%). Within this group, 31 patients (51.6%) experienced CVAE following the initiation of therapy with CFZ. Table 1 summarizes the demographic and clinical characteristics of these patients with or without CVAE. Notably, a significantly higher proportion of patients who developed CVAE had elevated levels of B-type natriuretic peptide levels (BNP) (>100 pg/mL) or NT-pro BNP (>125 pg/mL) (58.1% vs. 13.8%) (*p* = 0.0004), indicating potential pre-existing cardiovascular risk. The baseline demographic and medical histories were otherwise similar between the two groups (Table 1).

### 2.2. Identification of Differentially Expressed miRNAs at Baseline

**Figure 1:**
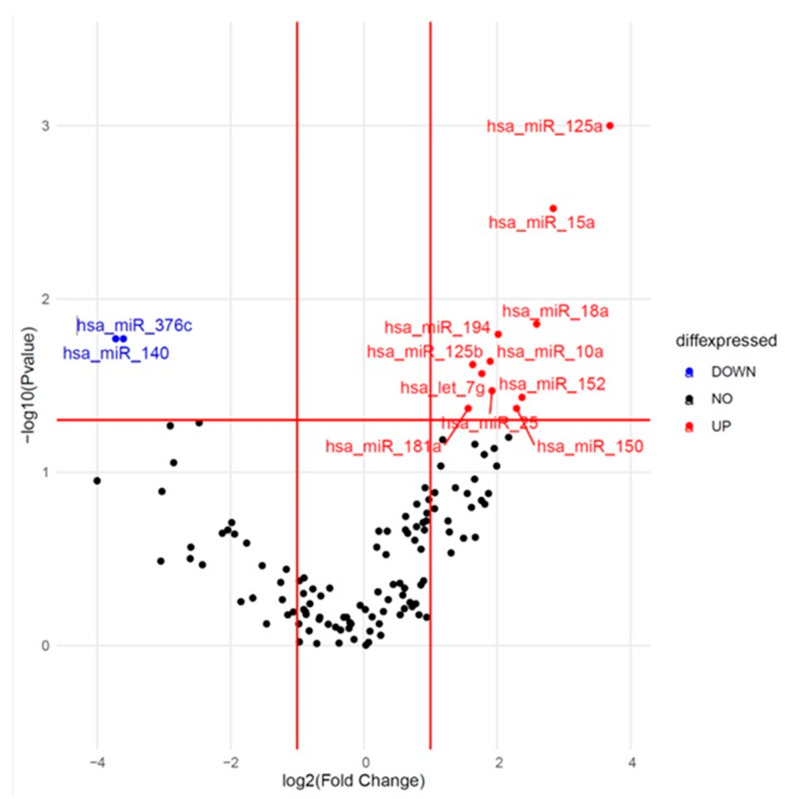
Volcano plots show differential expression of miRNA between CVAE and non-CVAE patients at the baseline. Volcano plot of significantly higher (red dots) and lower (blue dots). Negative log10 *p*-values are plotted on the y-axis, and log2 normalized fold change expression levels are plotted on the x-axis. A significant differential expression was detected with a *p* ≤ 0.05 and log2 fold change of >1.5 or <0.67.

**Figure 2:**
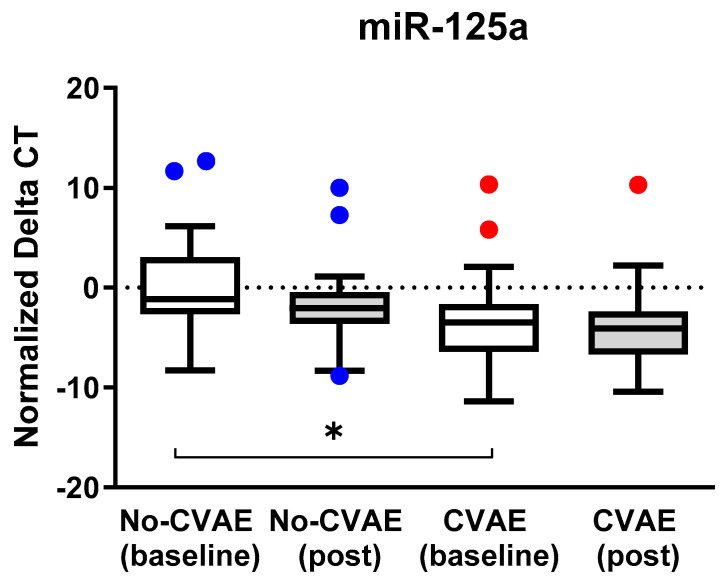
The expression of miR-125a at baseline and post-treatment in CVAE and non-CVAE patients. The normalized delta CT values are presented in box plots where the horizontal line in the middle of the box represents the median value. A lower delta CT value corresponds to a higher miRNA expression. The dots represent outliers with expressions lower than the 25th percentile—1.5xinterquartile range or higher than the 75th percentile + 1.5xinterquartile range. The blue dots represent the outliers in no-CVAE patients, and the red dots present outliers in the CVAE patients. * *p*-values comparing the CVAE and no-CVAE at baseline were 0.014 in the adjusted analysis. All other comparisons were not statistically significant.

One hundred twenty-five miRNAs passed quality control and were included in further analysis. We tested the association between the expression of these 125 miRNAs at baseline and the occurrence of CVAE. The results are presented as a volcano plot in Figure 1. Out of the 125 miRNAs, 13 showed consistent differential expression between the CVAE and non-CVAE groups at baseline before CFZ treatment. Specifically, 11 miRNAs had higher expression (Fold change (FC) > 1.5) and two had lower expression (FC < 0.67) in patients who later developed CVAE compared to those who did not develop CVAE in the unadjusted analysis (Table 2). Further analysis using logistic regression—adjusting for age, gender, race, and high BNP levels—identified significantly higher expression of miR-125a-5p, miR-15a-5p, miR-18a-3p, and miR-152-3p and a significantly lower expression of miR-140-3p at baseline in patients who later developed CVAE compared to those who did not (Table 2). Specifically, the relative expression level of miR-125a was significantly higher in the CVAE group compared to the non-CVAE group, with an FC of 12.9 (*p* = 0.0001). After adjusting for the covariates, the adjusted odds ratio (OR) was 1.25, with a 95% confidence interval (CI) of 1.05–1.48 (*p* = 0.014). However, there was no significant difference in miR-125a expression post-treatment between the two groups (*p* = 0.15) nor in the expression changes pre- and post-treatment (*p* = 0.55). The normalized delta CT values in CVAE and non-CVAE patients at baseline and post-treatment are illustrated in Figure 2 where lower delta CT values correspond to higher miRNA expressions.

### 2.3. Identification of Differentially Expressed miRNAs Post-Treatment

**Figure 3:**
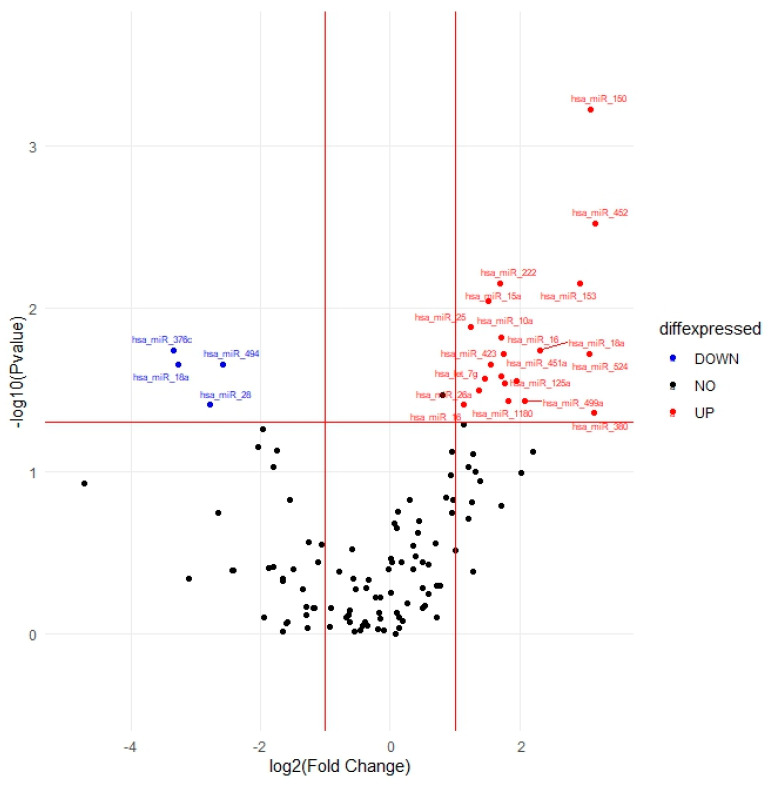
The plot illustrates the fold change and statistical significance of miRNA expression post-treatment. Volcano plots show differential expression of miRNA between CVAE and no-CVAE patients post-treatment. The miRNAs with significantly higher expression are shown in red dots and those with significantly lower expression are shown in blue dots. Negative log10 *p*-values are plotted on the y-axis, and log2 normalized fold change expression levels are plotted on the x-axis. A significant differential expression was detected with a *p* ≤ 0.05 and log2 fold change of >1.5 or <0.67.

The identification of differentially expressed miRNAs post-treatment is a crucial aspect of understanding the molecular alterations associated with the response to treatment in patients with MM. The expression patterns of these 125 miRNAs in CVAE vs. non-CVAE patients post-treatment are visually represented in Figure 3. Analyses of post-treatment samples identified 24 out of the 125 target miRNAs consistently differentially expressed in CVAE patients compared to non-CVAE patients. Among these, twenty miRNAs had higher expression, while four miRNAs had lower expression, in the CVAE patients compared to non-CVAE patients post-treatment in the unadjusted analysis (Table 3). After adjusting for the covariates, three miRNAs remained significant. The miR-150-5p showed significantly higher expression in CVAE patients compared to non-CVAE patients, with an OR of 1.31 and a 95% CI of 1.04–1.65 (*p* = 0.02, FC = 8.55). While miR-18a-5p and miR-494-3p had lower expression in the CVAE patients compared to non-CVAE patients (Table 3). To further illustrate the patterns of miRNA expression, we generated heat maps comparing the miRNA expression profiles between CVAE and non-CVAE groups at baseline and post-treatment. These heat maps provide a more intuitive visualization of the up-regulation and down-regulation patterns of miRNAs in both groups (Supplementary Figures S1 and S2).

### 2.4. Change in the miRNA’s Expression between Baseline and Post-Treatment

**Figure 4:**
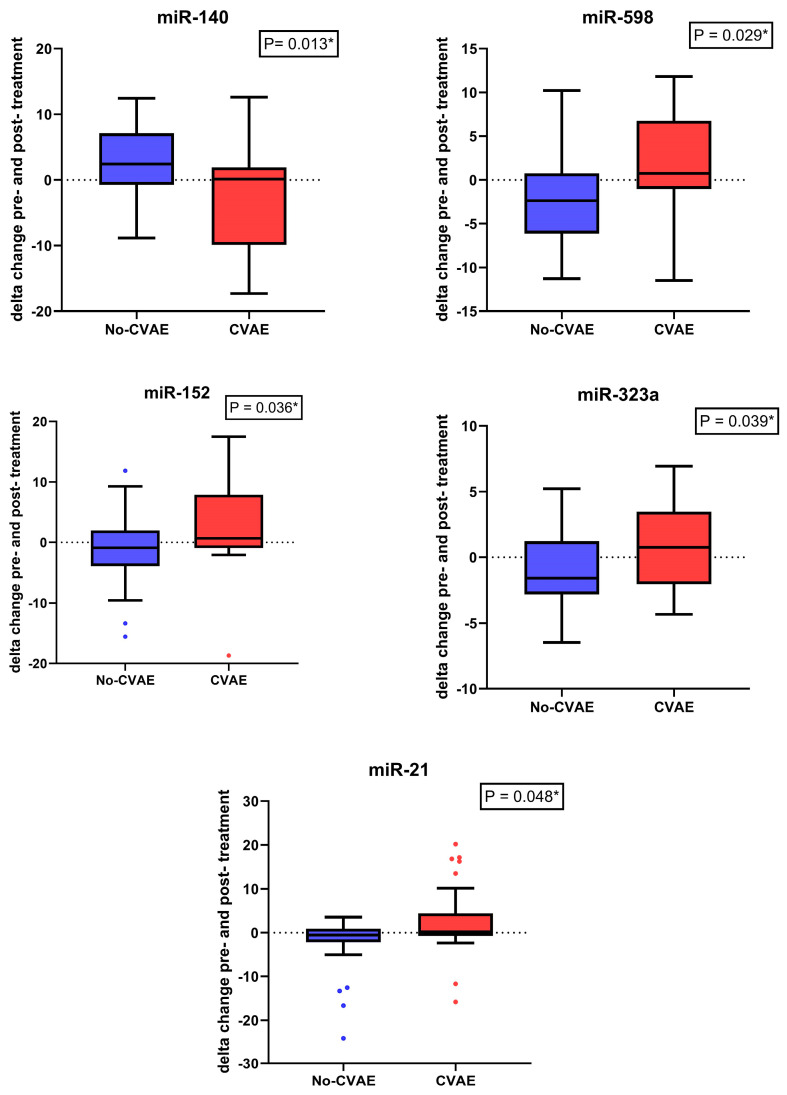
The change in the expression of miRNAs before and after carfilzomib treatment between patients with and with no CVAE. A lower delta CT value means a higher expression. * indicates these adjusted *p* values were <0.05. The blue color represents the non-CVAE group while the red color represents the CVAE group. The dots represent outliers with values lower than the 25th percentile—1.5 x interquartile range or higher than the 75th percentile + 1.5 x interquartile range. The blue dots represent the outliers in the no-CVAE group, and the red dots present outliers in the CVAE group.

**Figure 5:**
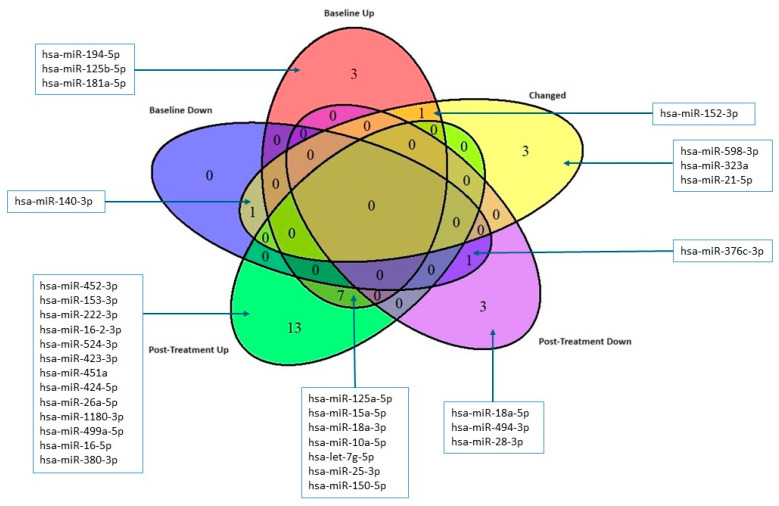
The Venn diagram illustrates the overlap between the miRNAs that are upregulated or downregulated at baseline and post-treatment and the miRNAs that were specifically affected by carfilzomib treatment. Red: Baseline Up; Blue: Baseline Down; Purple: Post-Treatment Down; Green: Post-Treatment Up; Yellow: Changed before and after treatment.

To assess the impact of CFZ treatment on miRNA levels, we performed a paired analysis of miRNA expression before and after treatment in both CVAE and non-CVAE patients. This analysis revealed significant differences in changes in miRNA expression following CFZ treatment. Five miRNAs responded significantly differently to CFZ treatment in CVAE patients compared to non-CVAE patients (Table 4). Notably, miR-140-3p had significantly lower expression at baseline in the CVAE patients compared to non-CVAE patients. However, in response to CFZ treatment, the expression of miR-140-3p in the CVAE patients was significantly elevated post-treatment compared to non-CVAE patients (Table 4). The change in the miR-140-3p expression as the result of CFZ treatment was significantly different in the CVAE patients compared to non-CVAE patients (*p* = 0.013, Figure 4). In contrast, several other miRNAs, including miR-598, miR-152, miR-21, and miR-323a exhibited significant decreases in expression levels after treatment in the CVAE patients compared to non-CVAE patients, as shown in Figure 4 (*p* ≤ 0.05). This differential expression highlights the dynamic changes in miRNA profiles in response to CFZ treatment, suggesting their potential role in mediating the biological effects of therapy. A Venn diagram summarizes the overlaps between the number of miRNAs that were differetially expressed at baseline and post-treatment and the number of miRNAs that had significantly different responses to CFZ treatment in CVAE and non-CVAE patients (Figure 5).

### 2.5. Pathway Enrichment Analyses

We performed miRNA enrichment analysis on the miRNAs that were significantly differentially expressed at baseline and post-treatment using the miEAA (miRNA Enrichment Analysis and Annotation) tool [29]. The top pathways (*p* < 10^−4^) enriched with the differentially expressed miRNAs between the CVAE and non-CVAE patients at baseline are summarized in Supplementary Table S1. Among these, the cysteine and methionine metabolism pathway (*p* = 7.45 × 10^−7^) was the most significantly over-represented pathway.

The top three pathways enriched with the miRNAs that were significantly differentially expressed post-treatment in the CVAE patients compared to non-CVAE patients were the PI3 kinase pathway (*p* = 6.16 × 10^−5^), TGF beta signalling pathway (*p* = 6.73 × 10^−5^), and the ubiquitin–proteasome pathway (*p* = 2.14 × 10^−5^) (Supplementary Table S2). The GO biological process analysis indicates that miR-125a and miR-150 positively regulate the endothelial cell surface expressed chemotaxis and apoptosis regulator (*ECSCR*) gene, with a *q*-value of 0.000984.

## 3. Discussion

This study is the first study to explore baseline circulating miRNAs as potential biomarkers for CFZ-CVAE in MM patients. Our analysis identified five miRNAs with significant differential expression in CVAE vs. non-CVAE patients at baseline. Among these, four miRNAs, including miR-125a-5p, miR-15a-5p, miR-18a-3p, and miR-152-3p, had higher expressions in the CVAE patients compared to non-CVAE patients. In contrast, one miRNA, miR-140-3p had lower expression in CVAE vs. non-CVAE patients at baseline. These identified miRNAs have the potential to be considered as baseline biomarkers for CFZ-CVAE. In addition, we identified three miRNAs (miR-150-5p, miR-18a-5p, and miR-494-3p) that were differentially expressed in patients with and without CVAE post-treatment of carfilzomib. Because we have both pre- and post-treatment data, our study is also the first study to evaluate miRNA changes in response to CFZ treatment. We identified five miRNAs, including miR-140-3p, miR-598-3p, miR-152-3p, miR-323a, and miR-21-5p, that responded differently to CFZ treatment among the patients who developed CVAE compared to those who did not. These miRNAs could provide insights regarding the underlying mechanisms of CFZ-CVAE.

The strongest baseline miRNA biomarker for CFZ-CVAE in our study is miR-125a-5p. We observed higher expression of miR-125a-5p in the baseline plasma of patients who later developed CZ-CVAE compared to those free of CVAE. The miRNA-125 family consists of miR-125a and miR-125b, which play a crucial role in the development of the cardiovascular system during the embryonic state [30,31]. They are also involved in the progression of various cardiovascular diseases, such as myocardial ischemia [32], atherosclerosis [33], ischemia-reperfusion injury [32], ischemic stroke [34], and HF [35]. Studies have shown that miR-125a is negatively associated with the long non-coding RNA lnc-ITSN1-2, which contributes to inflammation-related diseases. High lnc-ITSN1-2 expression increases inflammation and disease severity in acute ischemic stroke by suppressing genes that inhibit the anti-inflammatory effects of miR-107, miR-125a, and miR-146a, and by activating pro-inflammatory pathways such as NF-κB and TLR. [36]. As a result, pro-inflammatory markers like CRP, TNF-α, IL-1β, and IL-6 are upregulated, leading to inflammatory responses and enhanced disease severity in acute ischemic stroke patients. Additionally, lnc-ITSN1-2 may inhibit the anti-angiogenesis effects of miR-107, miR-125a, and miR-146a, contributing to vascular alterations and increased disease severity [36]. One of the key roles of miR-125a is its involvement in endothelial cell function and vascular health. For example, miR-125a can regulate angiogenesis (new blood vessel formation) by targeting and modulating the expression of important genes like related transcriptional enhancer factor-1 (RTEF-1), endothelial nitric oxide synthase (eNOS), and vascular endothelial growth factor (VEGF). This regulation is crucial for maintaining vascular health and can influence the development of conditions like atherosclerosis [31]. In a study of acute ischemic stroke and matched healthy control subjects, miR-125a-5p was found to be upregulated by 1.8-fold in those with ischemic stroke compared to controls [37]. These findings suggest that miR-125a-5p holds potential as a predictive biomarker for CFZ-CVAE.

In the post-treatment analysis, miR-150-5p was identified as the top miRNA that had significantly higher expression in the CVAE patients compared to non-CVAE patients (FC = 8.55). miR-150-5p was found to predict overt HF in patients with univentricular hearts [38]. A genome-wide prospective study found miR-150-5p to be significantly dysregulated in advanced HF patients and was associated with maladaptive remodeling, disease severity, and outcome [39].

GO analysis identified a statistically significant positive regulation of miR-125a and miR-150 by the *ECSCR* gene, with a compelling *q*-value of 0.000984, indicating a strong association with less than 0.1% likelihood of occurring by chance. This finding is crucial as *ECSCR* plays a pivotal role in endothelial cell functions, which are fundamental to vascular integrity and repair mechanisms [40]. The significance of this regulation extends beyond genetic interaction, as evidenced by clinical observations in our cohort of patients treated with CFZ. This correlation suggests that the interaction between ECSCR and these miRNAs may influence susceptibility to cardiovascular complications in a therapeutically stressed environment.

Our study also observed a significant elevation in miR-140-3p expression in response to CFZ treatment in CVAE patients compared to non-CVAE patients. The significance of miR-140-3p in cardiovascular pathophysiology underscores its involvement in key biological processes such as inflammation [41], cell apoptosis [42], and vascular remodelling. A previous study showed how circRNA_000203 affects cardiac hypertrophy by enlarging cell size and upregulating specific cardiac genes. It acts by sponging miR-26b-5p and miR-140-3p, preventing their usual inhibition of the Gata4 gene, thus increasing Gata4 expression and exacerbating hypertrophy [43]. Li and colleagues demonstrated that miR-140 was upregulated while mitofusin 1 (Mfn1) was downregulated during cardiomyocyte apoptosis induced by reactive oxygen species and doxorubicin [44]. Mfn1 is involved in the regulation of mitochondrial fusion and is able to inhibit mitochondrial fission and apoptosis in cardiomyocytes [45]. Li et al. also showed that miR-140 inhibits Mfn1 expression by targeting the 3′UTR, and the knockdown of miR-140 leads to a reduction in mitochondrial fission and apoptosis of cardiomyocytes [44]. The prognostic value of miR-140-3p variants in cardiovascular diseases has been substantiated by studies showing their potential as circulating biomarkers. Notably, miR-140-3p levels have been associated with cardiovascular mortality in patients with acute coronary syndrome, pointing to its utility in clinical risk assessment [46]. miR-140-3p was associated with cardiovascular death with a hazard ratio of 2.88 per standard deviation increase in expression among patients with coronary artery disease [47]. The modulation of miR-140 could, therefore, provide a novel approach to mitigating the risk of cardiovascular adverse events in at-risk patients. These insights into the role of miR-140-3p in cardiovascular adverse events post-treatment highlight the importance of integrating miRNA profiling into the clinical evaluation of cardiovascular risks associated with therapeutic interventions. Further investigations and validations of miR-140-3p as a biomarker and therapeutic target are essential for advancing cardiovascular disease management and treatment optimization.

Another miRNA that had a significant difference in changes in response to CFZ treatment between the CVAE and non-CVAE patients is miR-21-5p. A previous study demonstrated that miR-21 regulates the ERK–MAP kinase pathway in cardiac fibroblasts, affecting cardiac structure and function. In failing hearts, miR-21 increases in fibroblasts, boosting ERK–MAP kinase activity by inhibiting Spry1, which controls fibroblast survival, growth factor secretion, interstitial fibrosis, and cardiac hypertrophy. Silencing miR-21 in a mouse disease model reduces cardiac dysfunction and fibrosis, highlighting miR-21 as a potential therapeutic target for HF [48].

In the pathway analyses, the cysteine and methionine metabolism pathway was the most significant pathway that was enriched with differentially expressed miRNAs at baseline. This finding suggests that MM patients at high risk for CFZ-CVAE already differed from the patients without CVAE prior to CFZ treatment in terms of cysteine metabolism. Cysteine is synthesized in our body from methionine and plays a vital role in many processes, including the synthesis of essential fatty acids [49]. Recently, cysteine metabolism has been linked to mitochondrial respiratory function [50]. Since mitochondrial dysfunction has been increasingly recognized as an important contributing factor in the pathogenesis of HF [51], our finding is interesting. Further investigation is warranted to determine the link between the top baseline miRNAs and cysteine metabolism and mitochondrial dysfunction in patients at high risk for CFZ-CVAE.

Our pathway enrichment analysis of the significant miRNAs post-treatment indicated that these miRNAs were significantly over-represented in the PI3 kinase pathway, TGF-β signalling pathway, and the ubiquitin–proteasome pathway. The ubiquitin–proteasome pathway finding was not surprising given that CFZ targets this pathway and reduces proteasomal activity. Our finding of the PI3 kinase pathway appears to be consistent with the findings in a prior mouse study by Efentakis et al. [7]. This study demonstrated that CFZ cardiotoxicity is mediated through inhibition of the AMPKα pathway and inactivation of the PI3K/Akt/eNOS pathway, which plays pivotal roles in myocardial cell growth and survival. The TGF-β system stimulates myocyte hypertrophy and cardiac fibrosis. A study suggested the association of inflammation and TGF-β1-induced cardiac fibrosis in HF patients with preserved LVEF [52]. The fact that TGF-β signalling pathway showed up in our post-treatment pathway analysis was intriguing because most of the patients with CFZ-induced HF had preserved LVEF.

The identified miRNAs, particularly those showing significantly higher or lower expressions in CVAE patients compared to non-CVAE patients at baseline, may serve as promising candidates for further exploration as predictive biomarkers in the context of CFZ-CVAE. Our findings also shed light on specific miRNAs whose expression is notably altered in response to CFZ treatment, offering potential insights into the molecular mechanisms underlying CFZ-CVAE in MM patients. Further validation and mechanistic studies are warranted to elucidate the precise roles of these miRNAs in the observed differential expression patterns and their implications for cardiovascular health in MM patients undergoing CFZ treatment.

Even though this study is the first to assess the role of miRNA in the CFZ-CVAE, we recognized that there are some limitations. This study focuses on baseline miRNA expression and changes post-treatment, but it may not account for temporal fluctuations in miRNA levels during different stages of the disease and treatment, necessitating longitudinal studies to understand these dynamics better. The identified miRNAs with differential expression require validation in larger and independent cohorts to confirm their reliability as biomarkers. Additionally, while the study links specific miRNAs to CVAE, the precise biological mechanisms through which these miRNAs influence cardiovascular health remain to be fully elucidated, necessitating further functional studies to understand these pathways better.

## 4. Materials and Methods

### 4.1. Patients

This study included patients enrolled in the Prospective Observation of Cardiac Safety with Proteasome Inhibitor (PROTECT) study. PROTECT was conducted at Vanderbilt University Medical Center (VUMC) and the University of Pennsylvania Abramson Cancer Center (Penn) between September 2015 and March 2018 [28] and was designed to compare CVAE associated with CFZ or bortezomib-based regimens in MM patients. Patients with relapsed MM (defined according to the International Myeloma Working Group) [53] and receiving CFZ over 18 months were included in this study. Patients with symptomatic cardiac arrhythmia or New York Heart Association class 3 or 4 heart failure within three months before enrollment, as well as those with light chain amyloidosis identified through clinical history, ECG, transthoracic echocardiography (TTE), albuminuria, troponin elevation, end-organ biopsy, or, in select cases, cardiac magnetic resonance imaging, were excluded. A cardiologist assessed and graded cardiotoxicity in real time using the Common Terminology Criteria for Adverse Events (CTCAE) version 4.03 [54].

The main finding of the PROTECT study was that over a median follow-up period of 25 months, CVAE occurred in 51% of the patients treated with CFZ and 17% of those treated with bortezomib. Notably, patients with elevated B-type natriuretic peptide levels (BNP > 100 pg/mL or NT-proBNP > 125 pg/mL) before the initiation of CFZ-based therapy had a substantially higher risk of experiencing CVAE (Hazard Ratio 4.1, 95% CI 2.1–8.1, *p* < 0.0001) [28].

Plasma samples were collected from MM patients at baseline, before CFZ treatment initiation, and at every cycle afterward up to 12 months post-baseline. A cohort of 60 MM patients receiving CFZ was included in the analysis, comprising 31 patients who developed CVAE. For patients who developed CVAE, plasma samples obtained at the time of CVAE diagnosis were assessed as post-treatment samples. Given that all CVAE occurred within the six months of CFZ treatment, for patients who did not experience CVAE, the plasma samples at the six-month visit were used for analysis as post-treatment samples.

### 4.2. Circulating miRNA Isolation and Open Array Profiling

Total RNA extraction from 100 μL plasma samples was performed using the MagMAX mirVana Total RNA Isolation Kit (Applied Biosystems, Waltham, MA, USA). The TaqMan™ OpenArray Human microRNA panels on the QuantStudio™ 12K Flex platform (Applied Biosystems, Waltham, MA, USA) were used for the initial screening phase. This fixed-content panel consists of 754 miRNAs with validated human TaqMan™ MicroRNA assays obtained from Sanger miRBase release version 14.

Around 100 ng of total RNA was used for cDNA synthesis using Megaplex RT primer pools A and B and TaqMan™ MicroRNA Reverse Transcription Kit according to the manufacturer’s protocol. Subsequently, preamplification was carried out using TaqMan™ preamplification master mix with Megaplex preamplification primer pools A and B. The pre-amplified products were then diluted and combined in a 1:1 ratio with TaqMan™ OpenArray Real-Time PCR Master Mix before being loaded onto the 384-well OpenArray Sample Loading Plate. The AccuFill System automatically loaded the TaqMan™ OpenArray Human MicroRNA Panels, which were then subjected to polymerase chain reaction (PCR) cycling in the QuantStudio 12K Flex Real-Time PCR System.

### 4.3. Statistical Analysis of Open Array Data

In the quality control procedure, miRNAs with Cq-values exceeding 40 or those with an amplification score below 1.24 were excluded. To ensure robust normalization, we employed a global normalization approach [55]. The combination of miR-24 and miR-16 yielded a highly stable value of 0.005 and was selected as internal control. To identify microRNAs that were differentially expressed in samples with CVAE vs. non-CVAE, we applied the criteria of a fold change (FC) greater than 1.5 or less than 0.67, along with a *p*-value of ≤ 0.05. For this analysis, we employed the 2^−ΔΔCT^ method [56]. Finally, the data underwent log transformation and were represented as −∆∆CT to achieve a dataset with a normal distribution. MiRNA relative expression, along with demographic variables, were presented as mean ± standard deviation. GraphPad Prism (v.10, GraphPad Software, La Jolla, CA, USA) was utilized to generate boxplots and connected scatter plots. Differential expression analyses were conducted through linear model tests using R’s native functions (https://www.r-project.org/, accessed on September 2022). Paired analysis was performed to assess the changes in miRNA expression before and after treatment. Statistical significance was determined at *p*-values < 0.05. We utilized the Mann–Whitney U test to compare the miRNA expression between CVAE and non-CVAE patients. Logistic regression analyses were performed to estimate the odds ratios (ORs) and 95% confidence intervals, adjusting for age, gender, race, and the binary variable of BNP levels above normal (BNP > 100 pg/mL or NT-pro BNP > 125 pg/mL). Statistical analyses were performed using SAS v9.4 (Cary, NC, USA), IBM Corp’s Statistical Package for the Social Sciences SPSS v.26 (Armonk, NY, USA), or R software (R4.4.1) (Vienna, Austria). The miRNA–mRNA regulatory network was reconstructed using the ENCORI and miRDB databases. Functional enrichment analysis of the related miRNAs was conducted using the miRNA Enrichment Analysis and Annotation tool (miEAA) (Jena, Thuringia, Germany) [29] and gene ontology (GO) (Cambridge, UK) [57]. Heat maps illustrating miRNA expression in CVAE and non-CVAE groups at baseline and post-treatment were generated using the Multiple Experiment Viewer (MeV 4.9.0) (Cambridge, MA, USA) [58]. The data were normalized and clustered to highlight the differences in miRNA expression patterns.

## 5. Conclusions

In conclusion, we identified several miRNAs that showed differential expression at baseline (i.e., miR-125a-5p), post-treatment (i.e., miR-150-5p), or responded differently to CFZ in patients with CVAE compared to those without CVAE (i.e., miR-140-3p). The identified miRNA biomarkers may prove instrumental in advancing personalized approaches to cardiotoxicity risk stratification and understanding the underlying mechanisms of CFZ-CVAE, ultimately enhancing the overall care and outcomes of MM patients undergoing CFZ treatment.

## Figures and Tables

**Table 1:** Baseline characteristics of MM patients.

Characteristics	CVAEs (n = 31)	Non-CVAEs (n = 29)	*p*-Value
Age	67.16 ± 8.81	64.66 ± 9.51	0.29
Sex (Male)	23 (74.19)	22 (75.86)	0.88
Race			>0.99
White	27 (87.10)	25 (86.21)	
Black	4 (12.90)	4(13.79)	
Smoker	15 (48.39)	11(37.93)	0.41
NYHA			0.75
Class 1	18 (58.06)	18 (62.07)	
Class 2	13 (41.94)	11 (37.93)	
Hypertension	15 (48.39)	8 (27.59)	0.10
CVD (family history)	19 (61.29)	15 (51.72)	0.45
Diabetes mellitus	4 (12.90)	4 (13.79)	>0.99
Hypercholesterolemia	8 (25.81)	11 (37.93)	0.31
Thrombosis	6 (19.35)	4 (13.79)	0.73
Arrhythmia	6(19.35)	4 (13.79)	0.73
BNP above cutoff *	18 (58.06)	4 (13.79)	0.0004
Baseline LVEF	60 (8)	64 (10)	0.17

CVAE: cardiovascular adverse events; NYHA: New York Heart Association Classification of Heart Failure. CVD: cardiovascular disease. LVEF: left ventricular ejection fraction. * BNP above cutoff: the binary indicator of whether the BNP or NT-pro BNP was above the cutoff at diagnosis (100 pg/mL for BNP, 125 pg/mL for NT-pro BNP).

**Table 2:** The differentially expressed miRNA between CVAE and non-CVAE patients at baseline.

miRNA	Unadjusted *p*-Value	FC	Adjusted *p*-Value	OR	95% CI
**hsa-miR-125a-5p**	**0.001**	**12.91**	**0.014**	**1.25**	**1.05–1.48**
**hsa-miR-15a-5p**	**0.003**	**7.16**	**0.026**	**1.22**	**1.02–1.45**
**hsa-miR-18a-3p**	**0.014**	**6.02**	**0.03**	**1.22**	**1.02–1.46**
hsa-miR-194-5p	0.016	4.04	0.192	1.12	0.95–1.32
**hsa-miR-140-3p**	**0.017**	**0.08**	**0.047**	**0.87**	**0.75–1.00**
hsa-miR-376c-3p	0.017	0.08	0.074	0.89	0.79–1.01
hsa-miR-10a-5p	0.023	3.71	0.218	1.11	0.94–1.33
hsa-miR-125b-5p	0.024	3.10	0.188	1.16	0.93–1.44
hsa-let-7g-5p	0.027	3.41	0.07	1.20	0.99–1.46
hsa-miR-25-3p	0.034	3.79	0.156	1.14	0.95–1.36
**hsa-miR-152-3p**	**0.037**	**5.18**	**0.031**	**1.16**	**1.01–1.34**
hsa-miR-150-5p	0.043	4.89	0.362	1.08	0.91–1.28
hsa-miR-181a-5p	0.043	2.96	0.084	1.22	0.97–1.53

FC: fold change between CVAE and non-CVAE patients; CVAE: cardiovascular adverse event; OR: odds ratio; CI: confidence interval. Adjusted *p*-values presented were from multivariable logistic regression adjusting for age, gender, race, and BNP above the cutoff at diagnosis (100 pg/mL for BNP, 125 pg/mL for NT-pro BNP). Bolded are the miRNAs that remained significant in the adjusted analysis.

**Table 3:** The differentially expressed miRNA in CVAE compared to non-CVAE patients post-treatment.

Target Name	Unadjusted *p*-Value	FC	SE	Adjusted *p*-Value	OR	95% CI
**hsa-miR-150-5p**	**0.0006**	**8.55**	**0.117**	**0.020**	**1.31**	**1.04–1.65**
hsa-miR-452-3p	0.003	8.95	0.094	0.091	1.17	0.98–1.41
hsa-miR-153-3p	0.007	7.57	0.083	0.236	1.10	0.94–1.30
hsa-miR-222-3p	0.007	3.25	0.138	0.088	1.27	0.97–1.66
hsa-miR-15a-5p	0.009	2.87	0.083	0.117	1.14	0.97–1.34
hsa-miR-25-3p	0.013	2.37	0.115	0.124	1.19	0.95–1.50
hsa-miR-10a-5p	0.015	3.29	0.113	0.077	1.22	0.98–1.52
hsa-miR-18a-3p	0.018	5.00	0.092	0.056	1.19	1–1.43
hsa-miR-376c-3p	0.018	0.10	0.066	0.188	0.92	0.81–1.04
hsa-miR-16-2-3p	0.019	3.39	0.094	0.137	1.15	0.96–1.38
hsa-miR-524-3p	0.019	8.43	0.063	0.215	1.08	0.96–1.22
**hsa-miR-18a-5p**	**0.022**	**0.10**	**0.065**	**0.024**	**0.86**	**0.76–0.98**
hsa-miR-423-3p	0.022	2.95	0.075	0.344	0.93	0.80–1.08
**hsa-miR-494-3p**	**0.022**	**0.17**	**0.099**	**0.018**	**0.79**	**0.65–0.96**
hsa-miR-451a	0.026	3.28	0.08	0.251	1.10	0.94–1.28
hsa-let-7g-5p	0.027	2.75	0.104	0.179	1.15	0.94–1.41
hsa-miR-125a-5p	0.028	3.87	0.081	0.150	1.12	0.96–1.31
hsa-miR-424-5p	0.029	3.41	0.082	0.399	1.07	0.91–1.26
hsa-miR-26a-5p	0.032	2.59	0.095	0.132	1.16	0.96–1.39
hsa-miR-1180-3p	0.037	3.53	0.104	0.197	1.14	0.93–1.40
hsa-miR-499a-5p	0.037	4.22	0.102	0.075	1.20	0.98–1.47
hsa-miR-16-5p	0.039	2.20	0.094	0.137	1.15	0.96–1.38
hsa-miR-28-3p	0.039	0.15	0.079	0.161	0.89	0.77–1.05
hsa-miR-380-3p	0.044	8.83	0.064	0.183	1.09	0.96–1.23

FC: fold change; CVAE: cardiovascular adverse event; OR: odds ratio; CI: confidence interval. Adjusted *p*-values presented were from logistic regression adjusting for age, gender, race, and BNP above the cutoff at diagnosis (100 pg/mL for BNP, 125 pg/mL for NT-pro BNP). Bolded are the miRNAs that remained significant in the adjusted analysis.

**Table 4:** The miRNAs that responded differently to carfilzomib treatment between CVAE and non-CVAE patients.

miRNA	Baseline		Post-Treatment	
	FC	Relative Expression	FC	Relative Expression
hsa-miR-140-3p	0.08	Down	2.0	Up
hsa-miR-598-3p	3.50	Up	0.26	Down
hsa-miR-152-3p	5.18	Up	0.16	Down
hsa-miR-323a	2.93	Up	0.89	Down
hsa-miR-21-5p	4.51	Up	0.12	Down

FC: fold change.

## Data Availability

The raw data supporting the conclusions of this article will be made available by the authors upon request.

## Supplementary Materials

The following supporting information can be downloaded at: https://www.mdpi.com/article/10.3390/ijms25147795/s1.
