# Supplementary material for: Differential Expression of Circulating miRNAs and Carfilzomib-Related Cardiovascular Adverse Events in Patients with Multiple Myeloma
Source: Int J Mol Sci. 2024 Jul 16;25(14):7795. doi: 10.3390/ijms25147795 (PMC11276722; doi:10.3390/ijms25147795)
Supplement: Supplementary file 1 [file ijms-25-07795-s001.zip › ijms-3075241-supplementary.pdf]

Supplementary Data

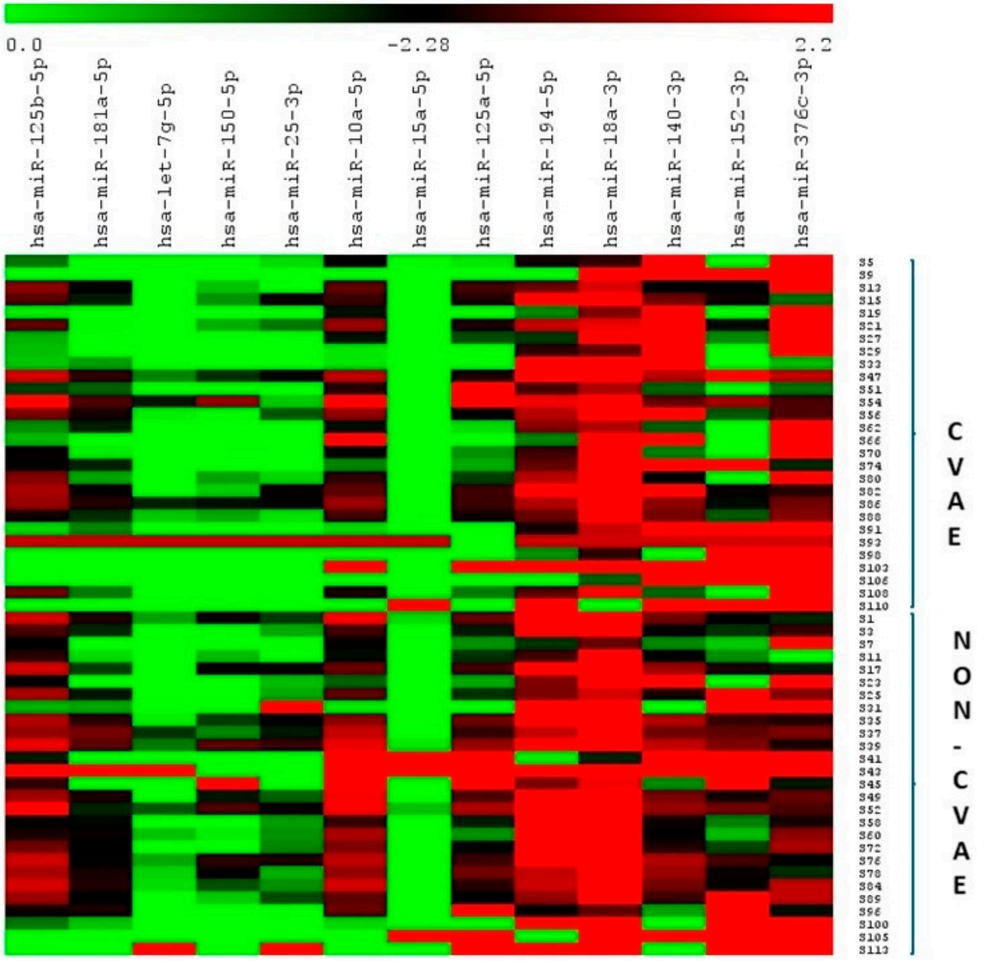

Supplementary Figure S1: Heat map for the miRNAs that are differentially expressed at baseline.

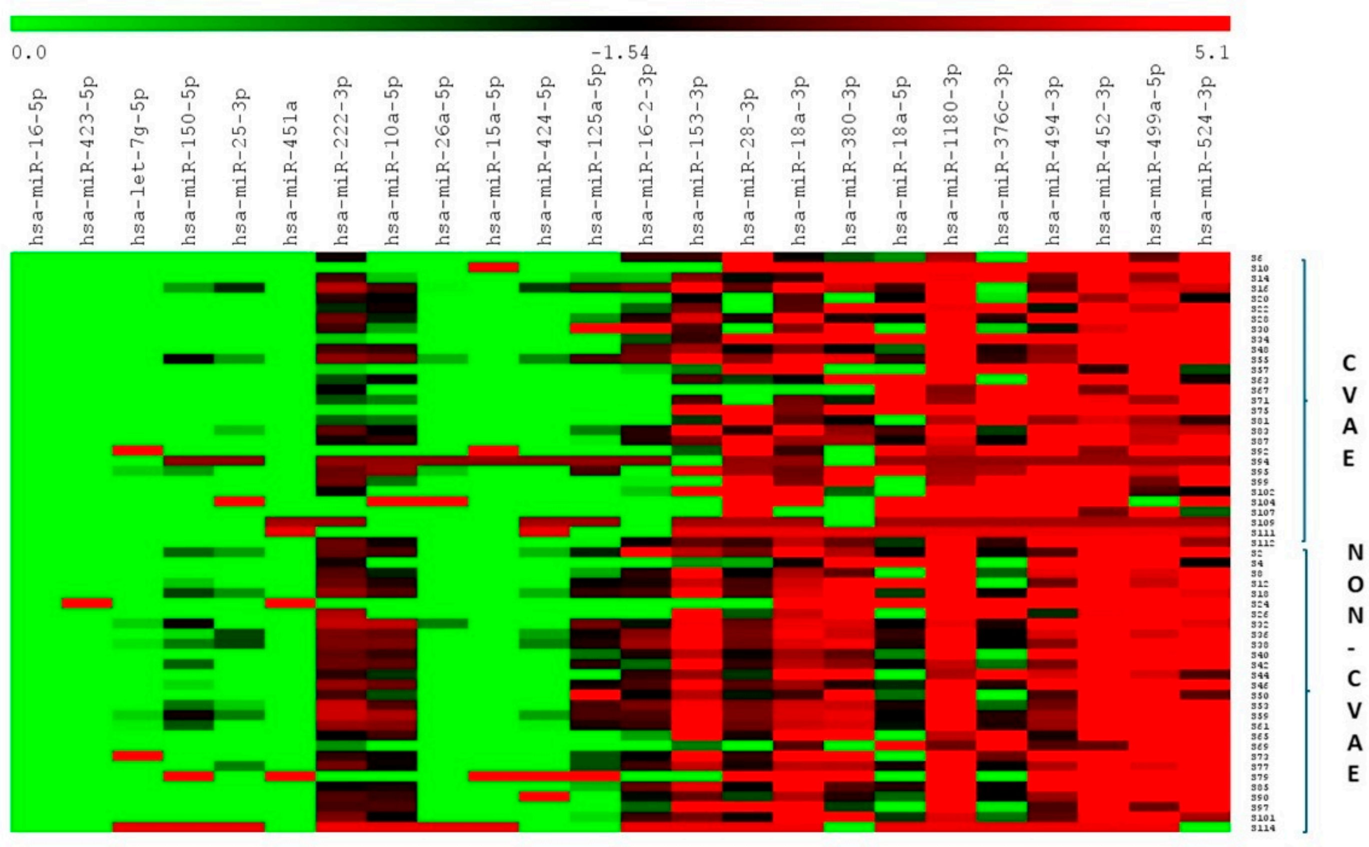

Supplementary Figure S2: Heat map for the miRNAs that are differentially expressed post-treatment.

Supplementary Table S1. Top pathways enriched for the significant miRNAs at baseline using the miRNA Enrichment and Annotation Analysis tool.

Category	Subcategory	Enrichment	P-value	P-adjusted	Q-value	Expected	Observed	miRNAs/precursors
Pathways (KEGG)	Cysteine and methionine metabolism	over-represented	7.45e-7	2.49e-4	2.49e-4	4.41126	13	hsa-miR-125a-5p; hsa-miR-15a-5p; hsa-miR-18a-3p; hsa-miR-194-5p; hsa-miR-140-3p; hsa-miR-376c-3p; hsa-miR-10a-5p; hsa-miR-125b-5p; hsa-let-7g-5p; hsa-miR-25-3p; hsa-miR-152-3p; hsa-miR-150-5p; hsa-miR-181a-5p
Pathways (miRWalk)	P00052 TGF beta signaling pathway	over-represented	3.33e-6	7.56e-4	7.56e-4	3.86214	12	hsa-miR-125a-5p; hsa-miR-15a-5p; hsa-miR-18a-3p; hsa-miR-194-5p; hsa-miR-140-3p; hsa-miR-376c-3p; hsa-miR-10a-5p; hsa-miR-125b-5p; hsa-let-7g-5p; hsa-miR-25-3p; hsa-miR-150-5p; hsa-miR-181a-5p
Pathways (miRWalk)	hsa04914 Progesterone-mediated oocyte maturation	over-represented	7.14e-6	7.56e-4	7.56e-4	4.11456	12	hsa-miR-125a-5p; hsa-miR-15a-5p; hsa-miR-18a-3p; hsa-miR-194-5p; hsa-miR-140-3p; hsa-miR-376c-3p; hsa-miR-10a-5p; hsa-miR-125b-5p; hsa-let-7g-5p; hsa-miR-25-3p; hsa-miR-152-3p; hsa-miR-181a-5p
Pathways (miRWalk)	hsa05210 Colorectal cancer	over-represented	7.32e-6	7.56e-4	7.56e-4	5.32621	13	hsa-miR-125a-5p; hsa-miR-15a-5p; hsa-miR-18a-3p; hsa-miR-194-5p; hsa-miR-140-3p; hsa-miR-376c-3p; hsa-miR-10a-5p; hsa-miR-125b-5p; hsa-let-7g-5p; hsa-

								miR-25-3p; hsa-miR-152-3p; hsa-miR-150-5p; hsa-miR-181a-5p
Pathways (miRWalk)	hsa05212 Pancreatic cancer	over-represented	7.32e-6	7.56e-4	7.56e-4	5.32621	13	hsa-miR-125a-5p; hsa-miR-15a-5p; hsa-miR-18a-3p; hsa-miR-194-5p; hsa-miR-140-3p; hsa-miR-376c-3p; hsa-miR-10a-5p; hsa-miR-125b-5p; hsa-let-7g-5p; hsa-miR-25-3p; hsa-miR-152-3p; hsa-miR-150-5p; hsa-miR-181a-5p
Pathways (miRWalk)	WP1601 Fluoropyrimidine activity	over-represented	1.05e-5	8.69e-4	8.69e-4	1.94369	9	hsa-miR-125a-5p; hsa-miR-15a-5p; hsa-miR-18a-3p; hsa-miR-194-5p; hsa-miR-10a-5p; hsa-miR-125b-5p; hsa-let-7g-5p; hsa-miR-25-3p; hsa-miR-150-5p
Pathways (KEGG)	Circadian rhythm	over-represented	7.24e-6	0.0012095	0.0012095	4.04535	12	hsa-miR-125a-5p; hsa-miR-15a-5p; hsa-miR-18a-3p; hsa-miR-194-5p; hsa-miR-140-3p; hsa-miR-10a-5p; hsa-miR-125b-5p; hsa-let-7g-5p; hsa-miR-25-3p; hsa-miR-152-3p; hsa-miR-150-5p; hsa-miR-181a-5p
Pathways (miRWalk)	WP1544 MicroRNAs in cardiomyocyte hypertrophy	over-represented	3.44e-5	0.0020366	0.0020366	3.71068	11	hsa-miR-125a-5p; hsa-miR-15a-5p; hsa-miR-18a-3p; hsa-miR-194-5p; hsa-miR-140-3p; hsa-miR-376c-3p; hsa-miR-10a-5p; hsa-miR-125b-5p; hsa-miR-25-3p; hsa-miR-152-3p; hsa-miR-181a-5p

Pathways (miRWalk)	WP254 Apoptosis	over-represented	3.45e-5	0.0020366	0.0020366	4.69515	12	hsa-miR-125a-5p; hsa-miR-15a-5p; hsa-miR-18a-3p; hsa-miR-194-5p; hsa-miR-376c-3p; hsa-miR-10a-5p; hsa-miR-125b-5p; hsa-let-7g-5p; hsa-miR-25-3p; hsa-miR-152-3p; hsa-miR-150-5p; hsa-miR-181a-5p
Pathways (miRWalk)	WP474 Endochondral ossification	over-represented	3.97e-5	0.0020385	0.0020386	3.76117	11	hsa-miR-125a-5p; hsa-miR-15a-5p; hsa-miR-18a-3p; hsa-miR-194-5p; hsa-miR-376c-3p; hsa-miR-10a-5p; hsa-miR-125b-5p; hsa-miR-25-3p; hsa-miR-152-3p; hsa-miR-150-5p; hsa-miR-181a-5p
Pathways (miRWalk)	WP481 Insulin signaling	over-represented	4.44e-5	0.0020385	0.0020386	4.79612	12	hsa-miR-125a-5p; hsa-miR-15a-5p; hsa-miR-18a-3p; hsa-miR-194-5p; hsa-miR-140-3p; hsa-miR-376c-3p; hsa-miR-10a-5p; hsa-miR-125b-5p; hsa-let-7g-5p; hsa-miR-25-3p; hsa-miR-152-3p; hsa-miR-181a-5p
Pathways (KEGG)	Drug metabolism–other enzymes	over-represented	2.46e-5	0.0022079	0.0022079	3.52697	11	hsa-miR-125a-5p; hsa-miR-15a-5p; hsa-miR-18a-3p; hsa-miR-140-3p; hsa-miR-376c-3p; hsa-miR-10a-5p; hsa-miR-125b-5p; hsa-let-7g-5p; hsa-miR-25-3p; hsa-miR-150-5p; hsa-miR-181a-5p
Pathways (KEGG)	Gap junction	over-represented	3.31e-5	0.0022079	0.0022079	5.89523	13	hsa-miR-125a-5p; hsa-miR-15a-5p; hsa-miR-18a-3p; hsa-miR-194-5p; hsa-miR-140-3p; hsa-miR-376c-3p;

								hsa-miR-10a-5p; hsa-miR-125b-5p; hsa-let-7g-5p; hsa-miR-25-3p; hsa-miR-152-3p; hsa-miR-150-5p; hsa-miR-181a-5p
Pathways (KEGG)	Notch signaling pathway	over-represented	3.10e-5	0.0022079	0.0022079	4.58405	12	hsa-miR-125a-5p; hsa-miR-15a-5p; hsa-miR-18a-3p; hsa-miR-194-5p; hsa-miR-140-3p; hsa-miR-10a-5p; hsa-miR-125b-5p; hsa-let-7g-5p; hsa-miR-25-3p; hsa-miR-152-3p; hsa-miR-150-5p; hsa-miR-181a-5p
Pathways (KEGG)	Pentose phosphate pathway	over-represented	4.14e-5	0.0023049	0.0023049	2.21071	9	hsa-miR-125a-5p; hsa-miR-15a-5p; hsa-miR-18a-3p; hsa-miR-376c-3p; hsa-miR-10a-5p; hsa-miR-125b-5p; hsa-miR-25-3p; hsa-miR-150-5p; hsa-miR-181a-5p
Pathways (miRWalk)	hsa04664 Fc epsilon RI signaling pathway	over-represented	5.60e-5	0.0023123	0.0023123	3.05437	10	hsa-miR-125a-5p; hsa-miR-15a-5p; hsa-miR-18a-3p; hsa-miR-194-5p; hsa-miR-140-3p; hsa-miR-10a-5p; hsa-miR-125b-5p; hsa-let-7g-5p; hsa-miR-25-3p; hsa-miR-181a-5p

Supplementary Table S2: Top pathways enriched for the significant miRNAs post-treatment using the miRNA enrichment and annotation analysis

Category	Subcategory	Enrichment	P-value	P-adjusted	Q-value	Expected	Observed	miRNAs/precursors
Pathways (miRWalk)	P00048 PI3 kinase pathway	over-represented	6.16e-5	0.0047112	0.0047112	6.19806	15	hsa-miR-153-3p; hsa-miR-222-3p; hsa-miR-15a-5p; hsa-miR-10a-5p; hsa-miR-18a-3p; hsa-miR-18a-5p; hsa-miR-423-3p; hsa-miR-494-3p; hsa-miR-451a; hsa-let-7g-5p; hsa-miR-125a-5p; hsa-miR-424-5p; hsa-miR-26a-5p; hsa-miR-499a-5p; hsa-miR-16-5p
Pathways (miRWalk)	P00052 TGF beta signaling pathway	over-represented	6.73e-5	0.0047112	0.0047112	6.23883	15	hsa-miR-150-5p; hsa-miR-222-3p; hsa-miR-15a-5p; hsa-miR-25-3p; hsa-miR-10a-5p; hsa-miR-18a-3p; hsa-miR-376c-3p; hsa-miR-18a-5p; hsa-miR-423-3p; hsa-let-7g-5p; hsa-miR-125a-5p; hsa-miR-424-5p; hsa-miR-26a-5p; hsa-miR-1180-3p; hsa-miR-16-5p
Pathways (miRWalk)	P00060 Ubiquitin proteasome pathway	over-represented	2.14e-5	0.0047112	0.0047112	3.54757	12	hsa-miR-222-3p; hsa-miR-15a-5p; hsa-miR-25-3p; hsa-miR-10a-5p; hsa-miR-18a-5p; hsa-miR-423-3p; hsa-let-7g-5p; hsa-miR-125a-5p; hsa-miR-424-5p; hsa-miR-26a-5p; hsa-miR-1180-3p; hsa-miR-16-5p
Pathways (miRWalk)	P04398 p53 pathway feedback loops 2	over-represented	7.94e-5	0.0047112	0.0047112	7.21748	16	hsa-miR-150-5p; hsa-miR-153-3p; hsa-miR-222-3p; hsa-miR-15a-5p; hsa-miR-25-3p; hsa-miR-10a-5p; hsa-miR-18a-3p; hsa-miR-18a-5p; hsa-miR-423-3p; hsa-miR-494-3p; hsa-miR-

								451a; hsa-let-7g-5p; hsa-miR-125a-5p; hsa-miR-424-5p; hsa-miR-26a-5p; hsa-miR-16-5p
Pathways (miRWalk)	WP1601 Fluoropyrimidine activity	over-represented	4.29e-5	0.0047112	0.0047112	3.13981	11	hsa-miR-150-5p; hsa-miR-222-3p; hsa-miR-15a-5p; hsa-miR-25-3p; hsa-miR-10a-5p; hsa-miR-18a-3p; hsa-miR-423-3p; hsa-let-7g-5p; hsa-miR-125a-5p; hsa-miR-26a-5p; hsa-miR-16-5p
Pathways (miRWalk)	WP2064 Neural crest differentiation	over-represented	8.35e-5	0.0047112	0.0047112	5.50485	14	hsa-miR-150-5p; hsa-miR-153-3p; hsa-miR-222-3p; hsa-miR-15a-5p; hsa-miR-25-3p; hsa-miR-10a-5p; hsa-miR-18a-3p; hsa-miR-18a-5p; hsa-miR-423-3p; hsa-miR-451a; hsa-let-7g-5p; hsa-miR-424-5p; hsa-miR-26a-5p; hsa-miR-16-5p
Pathways (miRWalk)	WP437 EGF–EGFR signaling pathway	over-represented	8.55e-5	0.0047112	0.0047112	8.23689	17	hsa-miR-153-3p; hsa-miR-222-3p; hsa-miR-15a-5p; hsa-miR-25-3p; hsa-miR-10a-5p; hsa-miR-18a-3p; hsa-miR-18a-5p; hsa-miR-423-3p; hsa-miR-494-3p; hsa-miR-451a; hsa-let-7g-5p; hsa-miR-125a-5p; hsa-miR-424-5p; hsa-miR-26a-5p; hsa-miR-1180-3p; hsa-miR-499a-5p; hsa-miR-16-5p
Pathways (miRWalk)	hsa05222 Small cell lung cancer	over-represented	8.55e-5	0.0047112	0.0047112	8.23689	17	hsa-miR-150-5p; hsa-miR-153-3p; hsa-miR-222-3p; hsa-miR-15a-5p; hsa-miR-25-3p; hsa-miR-10a-5p; hsa-miR-18a-3p; hsa-miR-16-2-3p; hsa-miR-18a-5p; hsa-miR-423-3p; hsa-miR-494-3p; hsa-miR-451a; hsa-let-7g-5p; hsa-miR-125a-5p; hsa-

								miR-424-5p; hsa-miR-26a-5p; hsa-miR-16-5p
Pathways (KEGG)	Apoptosis—multiple species	over-represented	3.28e-5	0.0064704	0.0064704	8.96013	19	hsa-miR-150-5p; hsa-miR-153-3p; hsa-miR-222-3p; hsa-miR-15a-5p; hsa-miR-25-3p; hsa-miR-10a-5p; hsa-miR-18a-3p; hsa-miR-376c-3p; hsa-miR-16-2-3p; hsa-miR-18a-5p; hsa-miR-423-3p; hsa-miR-494-3p; hsa-miR-451a; hsa-let-7g-5p; hsa-miR-125a-5p; hsa-miR-26a-5p; hsa-miR-499a-5p; hsa-miR-16-5p; hsa-miR-380-3p
Pathways (KEGG)	Carbohydrate digestion and absorption	over-represented	4.34e-5	0.0064704	0.0064704	5.60125	15	hsa-miR-150-5p; hsa-miR-222-3p; hsa-miR-15a-5p; hsa-miR-25-3p; hsa-miR-10a-5p; hsa-miR-18a-3p; hsa-miR-18a-5p; hsa-miR-423-3p; hsa-miR-494-3p; hsa-miR-451a; hsa-let-7g-5p; hsa-miR-125a-5p; hsa-miR-424-5p; hsa-miR-26a-5p; hsa-miR-16-5p
Pathways (KEGG)	Legionellosis	over-represented	5.81e-5	0.0064704	0.0064704	9.27912	19	hsa-miR-150-5p; hsa-miR-452-3p; hsa-miR-153-3p; hsa-miR-222-3p; hsa-miR-15a-5p; hsa-miR-25-3p; hsa-miR-10a-5p; hsa-miR-18a-3p; hsa-miR-376c-3p; hsa-miR-16-2-3p; hsa-miR-18a-5p; hsa-miR-423-3p; hsa-miR-494-3p; hsa-miR-451a; hsa-let-7g-5p; hsa-miR-125a-5p; hsa-miR-424-5p; hsa-miR-26a-5p; hsa-miR-16-5p
